# Genomic insights into antagonistic coevolution: collagen-like protein expansion and genome plasticity in the *Daphnia* parasite *Pasteuria ramosa*

**DOI:** 10.1093/g3journal/jkag091

**Published:** 2026-04-11

**Authors:** Alix Thivolle, Marjut Paljakka, Dieter Ebert, Peter D Fields

**Affiliations:** Department of Environmental Sciences, Zoology, University of Basel, Basel 4051, Basel-Stadt, Switzerland; Advance Executive Education, University of Jyväskylä, Jyväskylä 40014, Central Finland, Finland; Department of Environmental Sciences, Zoology, University of Basel, Basel 4051, Basel-Stadt, Switzerland; Department of Environmental Sciences, Zoology, University of Basel, Basel 4051, Basel-Stadt, Switzerland

**Keywords:** *Pasteuria ramosa*, *Daphnia magna*, host-pathogen interaction, collagen-like protein, genome rearrangement, genome assembly

## Abstract

Collagen-like proteins (CLPs) serve many pathogenic bacteria as adhesins to attach to host tissue. It was suggested that CLPs are a major determinant in the coevolution of the model system *Daphnia* (*Crustacean*) and its virulent pathogen *Pasteuria ramosa* (*Bacillota*). We analyzed 5 new high-quality genomes of *P. ramosa* originating from three host species. CLP genes underwent a radiation in *P. ramosa*. Genome comparison showed a high synteny, with few structural variations, mainly involving CLPs and transposases, both of which underwent recent multiplications. While most CLP genes are conserved, the presence/absence of some CLP genes varies among isolates. Our findings suggest a rapid radiation of CLP genes. These observations, derived from the first published genomes of *P. ramosa*, are consistent with the notion of rapid *Pasteuria*–*Daphnia* coevolution, as previously suggested for this highly specific host-parasite system.

## Introduction

Collagen is the major structural component in extracellular matrices of metazoans from sponges to humans (Vercellotti et al. 1985; Frantz et al. 2010; Qiu et al. 2021). In bacteria, the first collagen-like protein (CLP) was discovered in *Klebsiella pneumoniae* (Charalambous et al. 1988) and then found in other human-pathogenic species, such as *Streptococcus pyogenes* (Lukomski et al. 2000, 2001), *Bacillus anthracis* (Sylvestre et al. 2002), and *Legionella pneumophila* (Vandersmissen et al. 2010). Bacterial collagen proteins have a variety of functions related to host-pathogen interaction (Pizarro-Guajardo et al. 2014), among them the adhesion to host tissue (Srivastava et al. 2018), which is a prerequisite to colonize the host.

A compelling example of CLPs’ importance for host–pathogen interactions is found in the obligate parasite, *Pasteuria ramosa*, a virulent parasite of *Daphnia* water fleas, and a model system for studying host–parasite coevolutionary dynamics (Decaestecker et al. 2007; Andras et al. 2020; Ameline et al. 2022). A key feature of this interaction is the highly specific attachment of the parasite's transmission stages (=spores) to the host's cuticle, which follows a matching-allele model (Luijckx et al. 2011, 2013), where only specific combinations of host and parasite genotypes allow for successful attachment and, therefore, infection. A strong correlation between polymorphisms in a CLP gene, *Pcl7*, and the attachment phenotype was discovered (Andras et al. 2020) and later validated through a genetic manipulation study (Huessy et al. 2024). *Pcl7* is part of *P. ramosa*'s CLP gene family, which has an exceptionally high diversity, with about 50 genes (McElroy et al. 2011). This contrasts strongly with other pathogenic bacteria, which typically harbor three or fewer CLPs (McElroy et al. 2011). The extensive size of *P. ramosa's* CLP family suggests that other CLPs might also be involved in the attachment process of specific parasite strains to specific host genotypes. The diversity of *P. ramosa*–CLP genes is thought to be the mechanism that drives the high genotypic specificity of its infection in *D. magna* and may be the result of ancient host–pathogen coevolution (Bourgeois et al. 2021).

Up to date, no complete genome sequence is available for any *Pasteuria* species. Here, we produced the complete genomes of five *P. ramosa* isolates with different host infectotypes and geographic origins. Three isolates from *D. magna* represent the three *P. ramosa* lineages—phenotypic and genetic clusters—previously detected (Dexter et al. 2023). Two further isolates were sequenced that infect *D. longispina* and *D. dentifera*. A phylogenetic tree places *P. ramosa* firmly in the *Bacillota* phylum and reveals a monophyletic group formed with *P. penetrans* and *Thermoactinomyces* sp. We found 36 to 45 putative *P. ramosa-*CLP genes in each genome, alongside an unusually high number of transposases.

These genomic data will enable researchers to pinpoint the specific genes and mechanisms that govern the high-specificity infection process and provide a foundation for understanding the role of *Pasteuria* species as biocontrol agents for nematodes, another host where collagen is thought to be a key factor (Davies 2009; Kokalis-Burelle 2015).

## Material and methods

### *P. ramosa* system

*P. ramosa* (Bacteria: Bacillota) is an endospore-forming obligate parasite of the planktonic crustacean *Daphnia* first observed by Metchnikoff (Metchnikoff 1888). If the host and the pathogen genotype are compatible, attachment is possible. An attached bacterium penetrates the cuticle and enters the host's body cavity where it replicates, eventually producing millions of spores that are released after the death of the host. Infection results in host castration and a shortened lifespan (Ebert et al. 2016). Transmission is exclusively horizontal. Although *P. ramosa* is so far unculturable in vitro, it can be passaged repeatedly through susceptible hosts, and, as a result of the distinct bottleneck that takes place as part of the transmission process, pure lineages of single genotypes can be generated (Luijckx et al. 2011).

We produced high-quality DNA by focusing on the immature stage of the spores, which lack an exosporium and so can be lysed without causing DNA damage. Because immature spores are difficult to separate from the host tissue, we accounted for host DNA in the downstream analysis. We focus on five *P. ramosa* isolates. We chose one representative of 3 known phenotypic and genetic clusters of the parasite in *D. magna* called alpha, beta, and gamma lineages (Fredericksen et al. 2021; Dexter et al. 2023): *P. ramosa* isolate P21 (gamma lineage), C1 (beta lineage), and P54 (alpha lineage). We isolated P21 and P54 from infected *D. magna* individuals collected from the Aegelsee population in Switzerland (Switzerland, GPS: 47.558, 8.863), while C1 originated from a Moscow Zoo (Moscow, Russia, GPS: 55.763, 37.582) population (Luijckx et al. 2011). We further included isolate P10, which was isolated from an infected *D. longispina* in Finland (GPS location: 59.828755, 23.248008), and G18 from a *D. dentifera* isolated in North America [Midland Lake, Greene County, Indiana, USA (Auld et al. 2012)].

### Infection trials and collection of infected hosts

We maintained *Daphnia* under standardized conditions in artificial culture medium [ADaM, (Klüttgen et al. 1994)] at 20 °C. Infection trials followed a previously published protocol (Luijckx et al. 2014). We propagated the isolates C1 and P54 in the *D. magna* host clone HU-HO-2 (Bogarzo-to, Hungary, GPS: 46.8, 19.1333), P21 isolate in *D. magna* clone CH-H-t0_9.3_7 (Aegelsee), the P10 isolate in *D. longispina* (FI-FS-6-14) and the G18 isolate in *D. dentifera* (mid37).

At 14 d post-infection, once *P. ramosa* infections reached the grape-seed stage (the “immature spores”) (Ebert et al. 2016), we began collecting infected animals. Twelve hours before collection, we individually place infected *D. magna* into 100-mL jars with 50 mL of freshly filtered medium (ADaM, 0.2 µm filtered). Since *D. longispina* and *D. dentifera* are significantly smaller, we placed them in 24 well-plates with 1 mL of freshly filtered ADaM. *Daphnia* microbiota, undigested food, and the micro-biofilm covering the animal's carapace are significant sources of DNA contamination. To reduce DNA contamination from the gut content, we fed each animal dextran beads (Sephadex G-25 by Sigma Aldrich, 5 g/L) every 6 hours, a treatment that clears the gut. We also observed individuals every hour to see if they had molted, which happens every 3 to 4 days when *Daphnia* individuals shed and produce a new carapace not yet covered by a bacterial biofilm. We collected freshly molted individuals and pooled in a 100-mL jar containing freshly filtered ADaM and stored at 4 °C. We transferred batches of approximately 20 freshly molted individuals to a 1.5-mL microcentrifuge tube with 100 µL of G2 Buffer (lysis buffer from Genomic DNA Buffer Set, Qiagen ID: 19060) and carefully crushed with a plastic pestle less than 4 hours after their collection.

### DNA extraction and sequencing

We extracted DNA from P21-, P10-, G18-, and C1-infected animals using a Qiagen Genomic Tips kit (Qiagen ID: 10223). We sent P21 DNA sample to Genomic Facility of ETH D-BSSE (Basel, Switzerland) for sequencing where a PacBio SMRTbell library was prepared following DNA size selection with the BluePippin (DNA > 8 kb). Sequencing on a PacBio Sequel I SMRTcell yielded ∼5.1 Gbp of continuous long-read sequence data (∼2.4 million reads), with a mean subread length of 7.3 kb (N50 = 9.6 kb). For P10 and G18, the Lausanne Genomic Technology Facility performed sequencing on a single Pacbio Hifi SMRTcell (G18: 1,016,560 reads, mean read length: 10,307 bp. P10: 1,271,266 reads, mean read length: 10,404 bp).

We sequenced C1 genotype using the Oxford Nanopore MinION technology. We constructed the library for MinION using a ligation kit (SQK- LSK110, Oxford Nanopore Technology, Oxford, UK) and sequenced using two FLOMIN106 flow cells (v9.4.1), collecting in total 8,732,179 reads for a read length average of 562 bp. We basecalled the raw FAST5 data using Guppy v6.0.1 with the super accurate mode. We also used high-accuracy Illumina data, generating 17,875,920 Illumina MiSeq PE-250 bp reads based upon a Nextera XT DNA preparation kit. The Department of Biosystems Science and Engineering, ETH-Zurich in Basel, Switzerland, performed the sequencing.

For P54-infected animals, we extracted DNA following this protocol (https://www.protocols.io/view/dna-extraction-of-daphnia-and-symbionts-5jyl82n96l2w/v1). We constructed the library for MinION using a ligation kit (SQK- LSK114, Oxford Nanopore Technology, Oxford, UK) and sequenced using one FLOMIN114 flow cell (v10.4.1), collecting a total of 8.52 Gb, for a N50 read length average of 15.88 kb. We basecalled the raw pod5 data using dorado v 0.7.2 + 9ac85c6 with the super accurate mode (dna_r10.4.1_e8.2_400bps_sup@v5.0.0).

### Genome assemblies and annotations

#### P21 isolate assembly

We initially mapped all the reads to a draft genome of *P. ramosa* C1 based only on Illumina data (unpublished) and filtered out all the unmapped reads (236,507 mapped reads). We then used the Flye assembler (v2.8.3-b1695, Kolmogorov et al. 2019) with PacBio reads specific parameters to construct the primary assembly. To correct errors in the primary assembly, we used the Arrow pipeline from Genomic consensus toolkit to polish the genome (https://github.com/PacificBiosciences/GenomicConsensus). The assembled genome is 1.77 Mb and circular. To evaluate the relative biological completeness of this new genome draft, we used BUSCO v5.0.0 (Seppey et al. 2019) and the lineage database Firmicutes_odb10 (version 2021-02-23).

To ensure the absence of plasmids, we mapped the reads to a *Daphnia magna* genome of reference (Xinb3, PRJNA624896), filtered out the mapped reads, and assembled the reads using the Flye assembler (v2.8.3-b1695, Kolmogorov et al. 2019) with the meta option.

For the annotation, including gene prediction, tRNA and rRNA detection, we used Prokka v1.14.6 (Seemann 2014) with the whole bacterial non-redundant protein database from NCBI (RefSeq Release 204, 14 January 2021). We paid special attention to the annotation of the CLP genes. We downloaded the 38 known CLP genes sequences from the UniProt website and used this file as a reference set of trusted proteins to annotate with Prokka and the option “–protein”. In addition, Prokka gene predictions were examined with HMMER 3.3.2 (http://hmmer.org/, (Eddy 2011)) and a Pfam collagen domain (PF01391) to search for additional potential CLPs. We manually curated the list of CLPs and removed the fragments. We determined the origin of the chromosome with the web tool Ori-finder (March 2021, Luo et al. 2019).

#### C1, G18, P10, and P54 isolate assembly

We assembled C1 isolate using a hybrid approach that combined Oxford Nanopore Technologies (ONT) R9 reads and Illumina short reads, utilizing Unicycler v0.5.0 (Wick et al. 2017) as the assembly tool. In contrast, we assembled P10 and G18 genomes with Flye (Kolmogorov et al. 2020) from PacBio HiFi reads. Finally, we assembled P54 genome solely using ONT R10 reads. We applied the annotation pipeline initially employed for the P21 isolate to all the aforementioned genomes.

#### Transposase annotations

We annotated transposases using the webtool ISsaga (http://issaga.biotoul.fr/, Varani et al. 2011). We manually curated the list of putative transposases: to be considered as complete, a transposase should be composed of a DDE domain, annotated as IS5 or IS701, and be longer than 250 AA for IS5 and 300 AA for IS701. We excluded candidates with similarities to accessory genes, but without associated transposases, because we do not consider them as transposases. We aligned the proteins and calculated the pairwise identity using Clustal Omega (webtool 2022, Madeira et al. 2022).

### Phylogenetic tree and comparative genomics

To assess the phylogenetic relationship between the *Pasteuria* genus and the *Bacillota* phylum, we downloaded complete genomes and proteomes from a wide and representative sampling of *Bacillota* species (Supplementary Table 1), including a genome draft of the congeneric *P. penetrans* (Orr et al. 2018). As only the genome of *P. penetrans* was available, we annotated it using the same Prokka pipeline we used for *P. ramosa*. Other genomes used here are a subset of the data set used by (Antunes et al. 2016). We used BUSCO and the lineage Firmicutes_odb10 (version 2021-02-23) to assess genome completeness and to extract all 77 single-copy BUSCO genes present in all species. We aligned the single-copy using Prank v.170427 and concatenated with “catfasta2phyml.pl” script (https://github.com/nylander/catfasta2phyml). We ran PartitionFinder to determine the best partition (Stamatakis 2006; Kozlov et al. 2019), followed by RAxML-NG v1.1, that we ran according to this partition with the –bs-trees autoMRE option (Kozlov et al. 2019). The tree converged after 800 iterations. We clustered the proteomes using OrthoFinder (2.5.2) and functionally annotated using InterProScan (v5.52-86.0). We conducted COG annotation for each genome using eggNOG-mapper (webtool 2022, Cantalapiedra et al. 2021). We used Orthofinder on the subset containing the five isolates and *P. penetrans* to do comparative studies. We conducted a second single-copy BUSCO genes tree using the five *P. ramosa* isolates and *P. penetrans*, PartitionFinder find the models, and RAxML-NG used to create the tree, which converged after 200 iterations.

We aligned the CLPs using clustalo v1.2.4 with three iterations, and we produced the percentage of identity matrices produced for each CLP subfamily.

### Bacillota CLPs maximum likelihood tree

In order to detect CLPs, we performed a profile-based homology search of GXY motifs using the HMMER package with PF01391 motif (http://hmmer.org/, (Eddy 2011). We included only proteins longer than 150 AA and starting with a methionine. We aligned the 143 resulting CLP candidates with Prank v.170427, and we performed a Maximum Likelihood tree with RAxML-NG with the model PMB + IO + FO. The tree converged after 650 iterations.

## Results and discussion

### Genome metrics

**Fig. 1.**
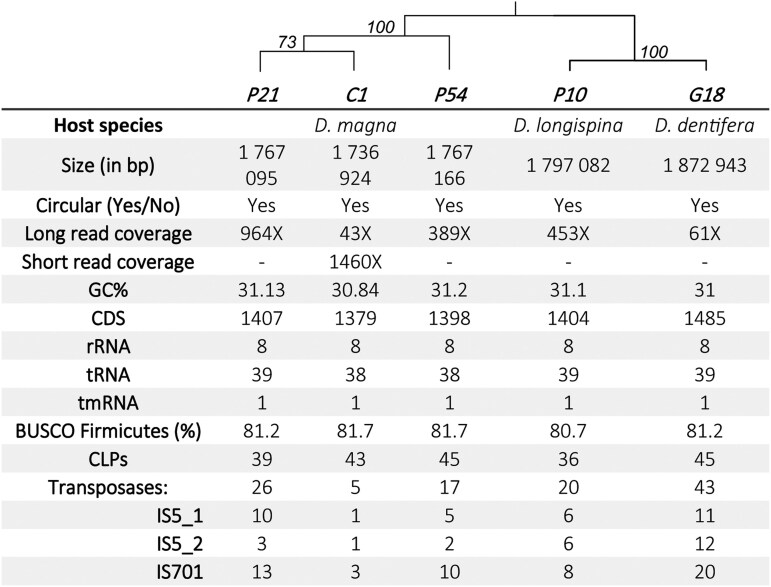
Genome metrics and schematic *Pasteuria ramosa* phylogenetic tree of the 5 isolates sequenced in this study. The phylogenetic tree was generated using single Busco genes and was rooted with *P. penetrans*. The value on the node is the bootstrap value and the tree converged after 200 iterations. Branch lengths are not representative.

The genome assemblies of the *P. ramosa* isolates resulted in single contigs of approximately 1.8 Mb, with 1,426-1,533 structural annotations (Fig. 1). In evaluating assembly completeness, we observed relatively low BUSCO scores (e.g. 81%) for the *Pasteuria* genus. Rather than indicating assembly or annotation gaps, we suggest these scores reflect significant genomic streamlining—a common evolutionary trajectory for specialized endoparasites (McCutcheon and Moran 2012, Supplementary File 1). This interpretation is supported by the successful assembly of the genomes into single, circularized contigs, and the absence of plasmids. For the following analysis, we used the P21 assembly as our focal genome. In this assembly, the 1,407 protein-coding sequences (CDS) represent 69.98% of the total genome (average length 878.9 bp), and the numbers are similar for the four other isolates (Fig. 1). For comparison, *P. penetrans,* a close relative, has 2,407 genes (average length 694 bp), representing 66.33% of the genome (Orr et al. 2018). The genomes of both *Pasteuria* species are less dense in coding regions than related species such as *Bacillus thuringiensis* (83.28%, (Lechuga et al. 2020)) or *Thermoactinomyces* sp. (86.56%, (Bezuidt et al. 2016)). The G + C content of all five *P. ramosa* isolates is around 31%, which is low compared to other *Bacillota* spp. (Lightfield et al. 2011) (*B. thuringiensis* 35%, *Thermoactinomyces* sp. 48%, *P. penetrans* 46%) (Orr et al. 2018). Neither a canonical CRISPR system nor prophages were identified in the *P. ramosa* genomes using CRISPRCasFinder (Couvin et al. 2018) and Phaster (Zhou et al. 2011; Arndt et al. 2016) tools.

### Phylogenetic position and phylogenetic structure of *P. ramosa*

**Fig. 2.**
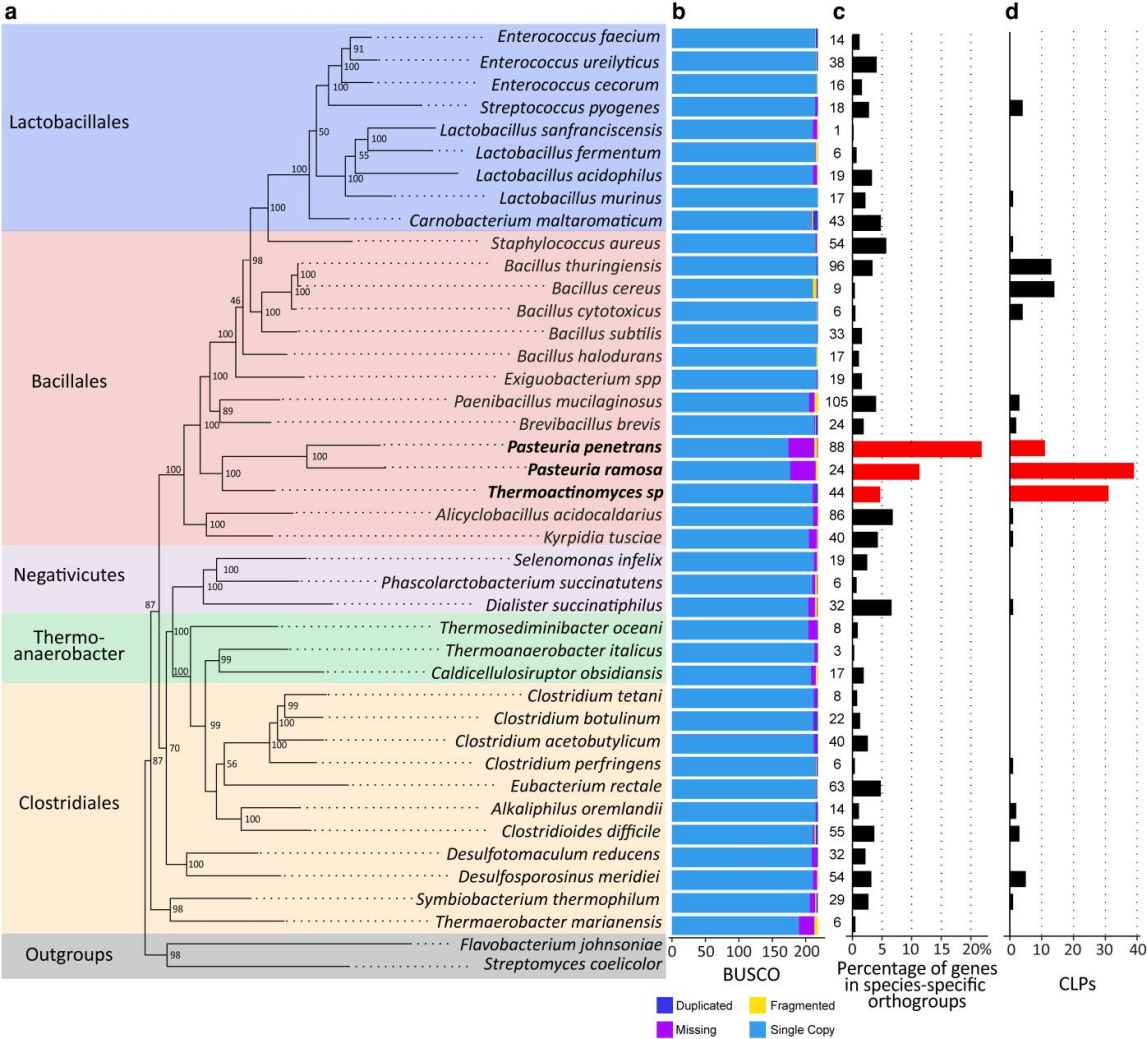
Phylogenetic tree and gene content comparison of selected *Bacillota* species. *P. ramosa* and *P. penetrans* have a high percentage of genes in species-specific orthogroups, while many BUSCO genes are missing. *P. ramosa* and *Thermoactinomyces* sp. have exceptionally high numbers of CLPs. Bar charts contain data for each species aligned to the corresponding species in the phylogenetic tree. a) The phylogenetic species tree was inferred using complete and single-copy BUSCO genes extracted from the entire genomes of all species shown here (*n* = 77). Bootstrap values (percent) are indicated at the node (800 iterations). Color bars on the left indicate bacterial orders. b) BUSCO gene counts. The reference database for BUSCO (v 5.0.0) was “firmicutes_odb10” with 218 core genes in total. The different portion of the bar refer to the number of complete single-copy orthologs, the complete duplicated orthologs, the fragmented or incomplete orthologs and the missing orthologs. c) Percentage of genes in species-specific orthogroups. Numbers on the left of the axis are the absolute numbers of genes in species-specific orthogroups. d) Number of CLPs in each species. *P. ramosa*, *P. penetrans*, and *Thermoactinomyces* sp. are highlighted, form a monophyletic group. The tree and data for *P. ramosa* are based on the genome of the P21 isolate.

A single-copy-BUSCO-gene phylogenetic tree based on the P21 genome confirms the *Pasteuria* placement within the phylum *Bacillota* and the class *Bacillales* (Fig. 2a, Supplementary Table 1). The tree shows that the *Pasteuria* genomes diverge deeply within the larger *Bacillales*, with the closest related clade including *Thermoactinomyces* sp. (Figure 2a).

*P. ramosa* and *P. penetrans* exhibit the lowest BUSCO score in the *Bacillota* phylum (81.2 and 80.3%, firmicutes_odb10, Fig. 2b), missing 38 and 39 highly conserved genes of the *Bacillota* phylum, respectively. *P. ramosa* and *P. penetrans* exhibit a high percentage of genes (11 and 21.8% respectively) in species-specific orthogroups (Fig. 2c, Supplementary File 1). *P. ramosa* has 24 species-specific orthogroups containing a total of 159 genes; the largest orthogroup contains 30 proteins annotated as transposases (Supplementary File 1).

Our *P. ramosa* were isolated from 3 different host species from two continents. Two host species, *D. longispina* and *D. dentifera*, belong to the subgenus *Daphnia Daphnia* (= *Daphnia* s.s.), while *D. magna* belongs to the subgenus *Daphnia Ctenodaphnia*. The subgenus *Daphnia Daphnia* and *Daphnia Ctenodaphnia* are believed to have diverged 145 million years ago (Cornetti et al. 2024). Phylogenetic analysis of *P. ramosa* (Fig. 1) suggests that isolates cluster according to host taxa (sub-genus)—*Ctenodaphnia* and *Daphnia* s.s.—rather than geographic origin. Notably, the *Daphnia* s.s. isolates remain closely grouped despite their intercontinental distribution.

### Collagen-like genes

Successful infection by *P. ramosa* is fundamentally dependent on the specific attachment of spores to the host cuticle. CLPs have been suggested to act as a bacterial adhesin, playing an essential role in the attachment of *Pasteuria* spores to its host are hypothesized to be the molecular mediators of the coevolutionary arms race between the parasite and *Daphnia* (Davies 2009; Andras et al. 2020). First discovered by Mouton et al. (2009), the *Pasteuria*-CLP repertoire is now known to be composed of more than 50 genes classified in 5 different groups (McElroy et al. 2011). Groups 1, 2, 3, and 4 form tight, sequence-specific clusters, while remaining CLPs are more divergent, non-clustering genes (McElroy et al. 2011). Crucially, many CLPs are organized into triplets, which consist of three CLPs—one each from groups 1, 2, and 3—arranged in a specific linear order and sharing the same transcriptional orientation. CPLs of groups 4 and the non-clustering CLPs do not exhibit a recognizable genome organization.

**Fig. 3.**
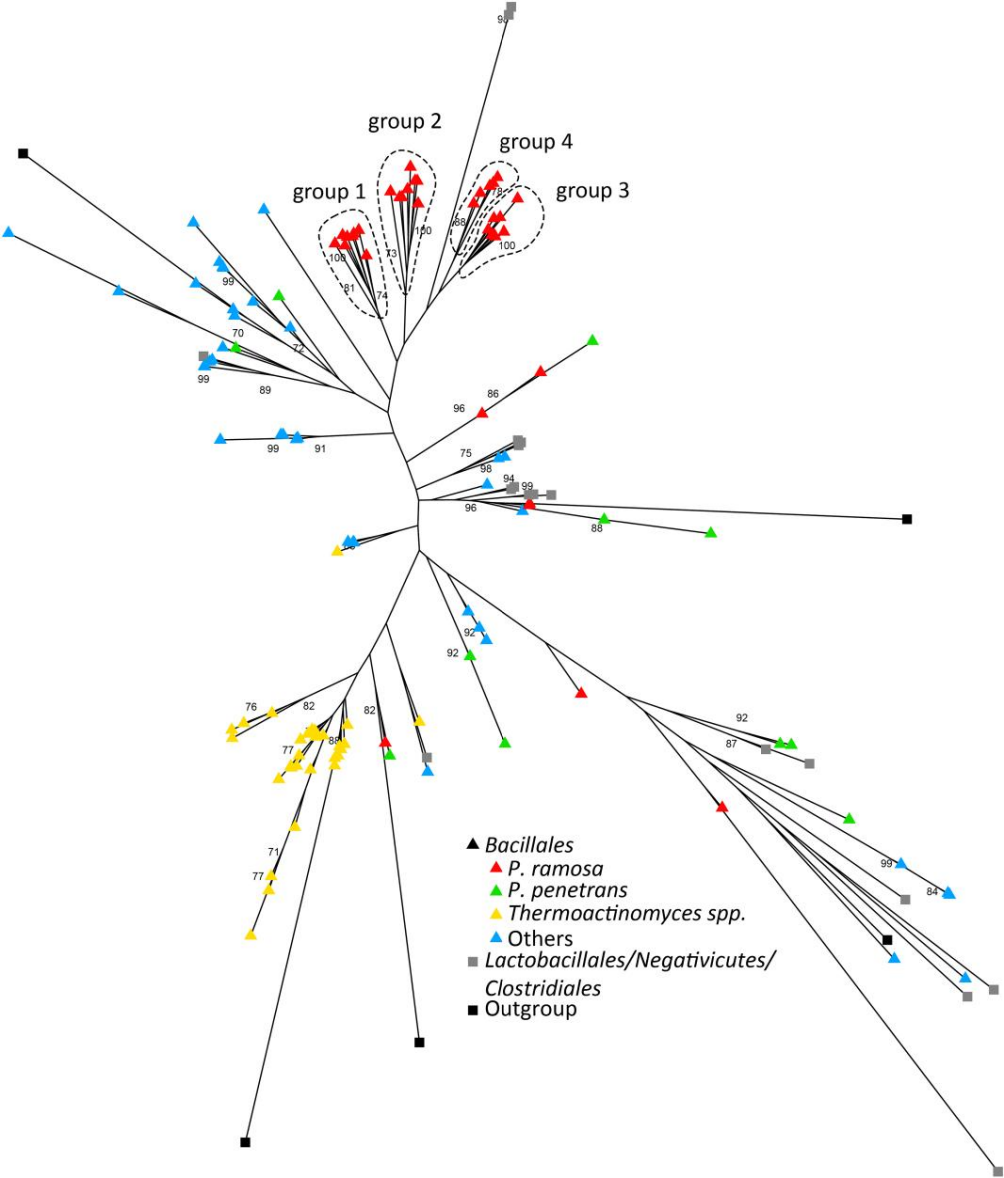
Gene tree of CLPs from the *Bacillota* species. Maximum-likelihood tree based on CLP gene sequences in the *Bacillota* species included in Fig. 2. While *P. ramosa*-CLP genes and *Thermoactinomyces sp.*-CLP genes are strongly clustered, the CLP genes of the other species, including *P. penetrans*, do not show a clear clustering pattern. Four clusters of *P. ramosa* CLP genes (group 1 to 4), correspond to the four groups previously found (McElroy et al. 2011). Genes from members of the Bacillales order are indicated by triangles; the other orders, by squares. Bootstrap support values over 70 are displayed on the nodes. The tree and data for *P. ramosa* are based on the genome of the P21 isolate.

Within the *Bacillota* phylum, CLPs are found predominantly in the *Bacillales* order (Qiu et al. 2021; Picker et al. 2022) with large numbers of them located mainly, though not exclusively, in the *Pasteuria/Thermoactinomyces* clade (Fig. 2d). Our phylogenetic analysis of all identified *Bacillota* CLPs shows that *P. ramosa* genes form a tight cluster with a distinct sub-structure of four groups (Fig. 3, groups1, 2, 3, and 4), a pattern consistent with previous findings and suggesting a clear radiation of these CLPs from a single ancestor for each group (McElroy et al. 2011). We also observed some *P. ramosa*-CLPs (no group) scattered throughout the tree, suggesting they are older and have a distinct evolutionary history from the radiating CLP groups.

Interestingly, we identified 47 hypothetical CLP genes in *Thermoactinomyces* sp. DSM 45891 that cluster almost perfectly together (with one exception, yellow in Fig. 3), indicating also here a species-specific radiation. While the massive CLP expansion is found in both the parasitic *P. ramosa* and the free-living thermophile *Thermoactinomyces* sp., the highly structured triplet organization or any specific organization seems to be unique to *P. ramosa*, suggesting antagonistic coevolution as the selective driver of the triplet architecture. It is important to note that two species of *Thermoactinomycetaceae*, *Baia soyae,* and *Croceifilum oryzae* are presenting this CLP expansion. While the *Thermoactinomyces* sp. DSM 45891 is poorly described, *B. soyae* is an endophytic bacterium of soja root (Guan et al. 2015), and *C. oryzae* originates from rice paddy soil (Hatayama and Kuno 2015). Most of the other species present a small number or none of CLPs (Supplementary File 2). Thus, the expansion of CLPs in *Thermoactinomycetaceae* is limited to certain species within this clade. This CLP expansion in *B. soyae* could be linked to its endophytic lifestyle and its need to adhere to the root to penetrate. For the two other species, it is not possible to formulate a hypothesis. Contrary to the two other species in the monophyletic group, we couldn’t detect any expansion of CLP in the *P. penetrans* genome, although the lower quality of the available genome may influence this.

An extensive search for CLP genes in each genome yielded a total of 39 CLPs in P21 and 43, 45, 36, 45 CLP genes in C1, P54, P10, and G18. The five genomes shared 30 of their CLPs (Supplementary Table 2), while other CLPs were unique to specific genomes and others had undergone duplication. A large proportion of CLPs are arranged in triplets and triplets show multiple duplication with variable degrees of identity: for example, in isolate P21*, Pcl6-7-8* is perfectly duplicated with one point mutation in *Pcl8*. In the genome of isolate G18, we detected three copies of the triplet *Pcl21-22-23*, with only gene *Pcl23* being identical, while *Pcl21* and *Pcl22* differ in sequences across copies (Supplementary Table 3). Finally, in the P54 genome, we detected the duplication of *Pcl58-59-60*. However, their sequence divergence suggests that they originated longer ago. These duplications and divergences are consistent with the hypothesis that all CLP triplets originate from a single ancestral CLP triplet (McElroy et al. 2011), suggesting that copy/paste mechanisms may be involved in the evolution of these triplets. While some CLP triplets exhibit duplication and diversification, one CLP in group 4, *Pcl32*, is conserved across all isolates (Supplementary Table 3).

In summary, the extensive duplication and divergence of the CLP triplets, possibly via copy-paste mechanisms, may reflect the signature of the ongoing coevolutionary interaction with its host, resulting in radiation and diversification. This radiation event took place before the genetic split of the host-associated *P. ramosa* clusters, as shown by the shared ancestral catalog of CLPs in all 5 isolates.

### Genome plasticity and synteny of the five isolates

The largest orthogroup specific to *P. ramosa* is annotated as containing transposases. Transposases play a role in genome rearrangement by promoting inversions, duplications, deletions, and transpositions. These changes can affect significant portions of genomes and may alter the infectivity or pathogenicity of pathogenic bacteria (Seferbekova et al. 2021).

43, 26, 20, 17, and 5 complete transposases have been detected in G18, P21, P10, P54, and C1, respectively, belonging to two families: IS701 and IS5 (Fig. 1). G18, P10, and P21 genomes exhibit a higher number of transposases with duplication or high identity between genes, suggesting more recent duplication or burst events (Supplementary Table 4). In contrast, none of the complete transposases in the C1 genome are identical. IS701 is absent from *P. penetrans* and *Thermoactinomyces*, while IS5 is present. *Thermoactinomyces* has a limited number of transposases, but *P. penetrans* also has a high number of transposases (Orr et al. 2018).

**Fig. 4.**
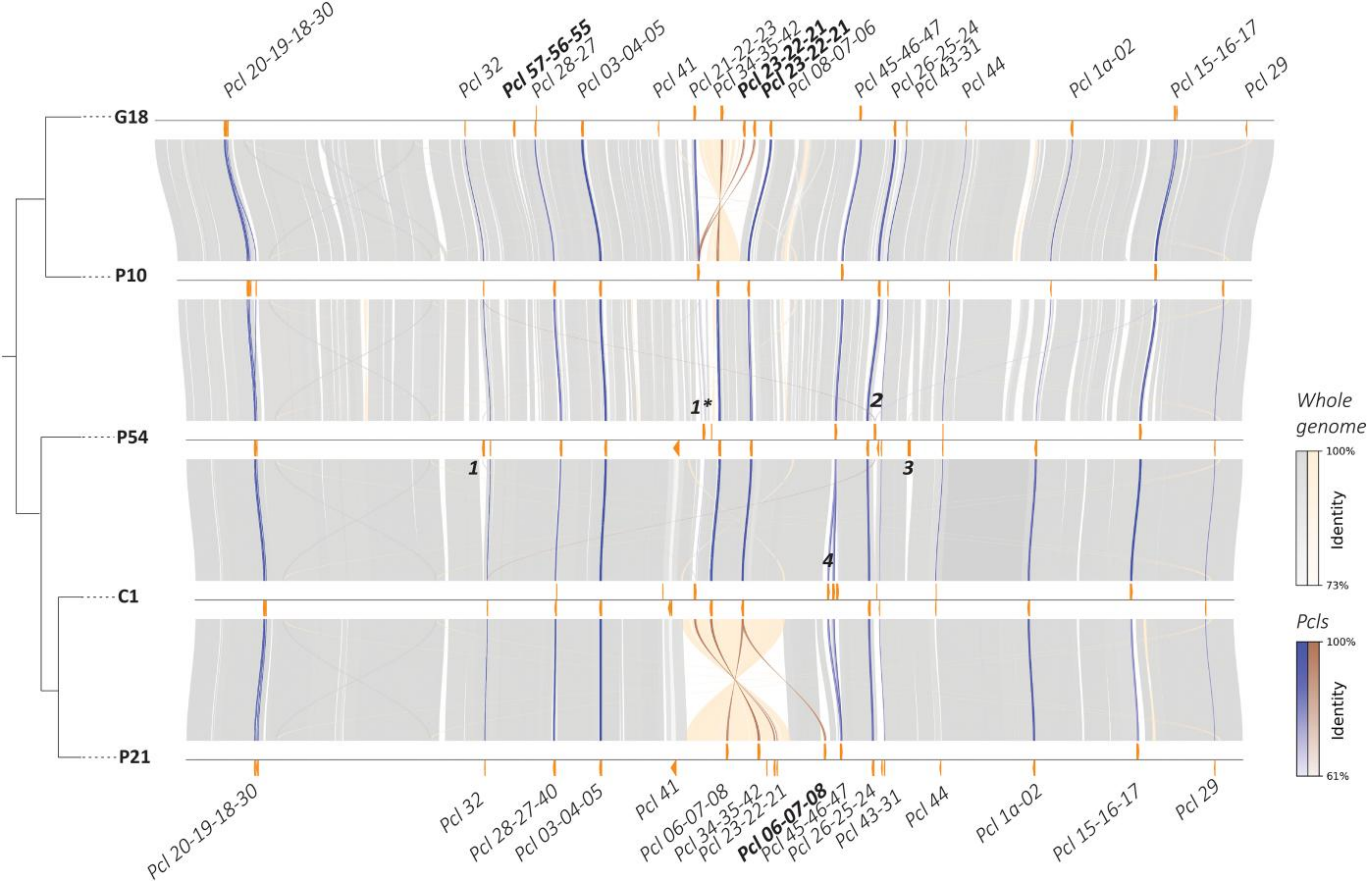
Synteny plot of the 5 different isolates: G18, P10, C1, P54, and P21. The phylogenetic tree was generated using single *Busco* genes and was rooted with *Pasteuria penetrans*, the tree converged after 200 iterations. Branch lengths are not representative. The right panel displays a synteny plot generated with Pygenomeviz (Shimoyama 2024). Only syntenic regions larger than 1.5 kb are shown. Homologous regions with an identity greater than 73% are represented in gray if the orientation is conserved between isolates and in colour if the orientation is inverted. Regions with lower identity or below the size threshold are excluded from the plot. CLPs are indicated on the top (and with tick marks) and shown in darker shadings, depending on their orientation, and their identity ranges from 35% to 100%. The numbers are the following CLPs: 1. Pcl 58-59-60, * indicate the replacement of the *Pcl21-22-23, 2. Pcl 55-56-57, 3. Pcl 52-53-54* and finally *4. Pcl 36-37-38/14-13-12/11-10-09.* Bolded *Pcl* triplet names identify triplets not situated in genomic loci where they are usually found.

To investigate the conservation of genome structure between the five isolates, we constructed a synteny plot (Fig. 4). This analysis revealed a large structural variant in the P21 genome: a fragment, 174 kb in size, is inverted in P21 compared to P54 and C1 (Fig. 4). This inversion contains 3 CLP triplets (*Pcl6-7-8*, *Pcl34-35-42*, *Pcl23-22-21*). In the same region, a smaller inversion is also present in the G18 genome (Fig. 4). Other than these two inversions, the genome synteny across the five isolates, as well as the genome identity, is high, even between isolates infecting different host species and coming from different continents.

Although the complete CLP catalog varies between isolates, those shared across the isolates occupy conserved genomic loci. One exception is the non-duplicated CLP triplet *Pcl55-56-57*, occupying two distinct regions in the genomes of G18 and P54 (Fig. 4, in bold in G18 and 2 in P54). In isolate P54, the *Pcl58-59-60* triplet is duplicated; one copy occupies the genomic locus where the *Pcl21-22-23* triplet is typically found in all other isolates (Fig. 4, 1*). Similarly, the triplet *Pcl06-07-08* is duplicated in P21 and found both its conserved site and at an additional locus. Finally, the triplet *Pcl21-22-23* is triplicated in G18. Despite the overall conservation of CLP positions, these gene duplications appear to result in the relocation of duplicated CLPs to new genomic regions.

Generally, a genome with a burst of transposases is consistent with the idea that this genome has recently undergone a host-restricted lifestyle (Siguier et al. 2014). Since *P. ramosa* and *D. magna* have been coevolving for millions of years, the transposases might play a role in the long-term coevolutionary process of this host–parasite system. We speculate that they have played an important role in the continuous diversification of the CLP family, a pattern that is consistent with the variation in the number of transposases from one isolate to another. Such a pattern could result in a constant birth and death process of genes, leaving behind a graveyard of non-functional genes and gene fragments. Indeed, in C1, we find many fragments and pseudogenes of transposases: out of 20 putative IS701 genes, 10 are only fragments, and 10 are non-functional proteins due to mutations. Note that we do not focus on the pseudogenisation of other gene families in the present study. The presumably high activity of transposases might also explain the low gene density of the *P. ramosa* genome, which is low compared to the 85–90% seen in free-living bacteria. This finding is comparable to other obligate parasites. In addition, the graveyard of transposases creates repetitive sequences in the genome that could be used as a template for repair or lead to replication errors.

## Conclusion

The *Daphnia—Pasteuria* system is a well-known model system for the study of long-term coevolution, which results in balancing selection of genes responsible for resistance in the host and infectivity (i.e. attachment) in the parasite. The *de novo* assembly of five complete *P. ramosa* genomes reveals a conserved genome architecture. Despite significant genomic streamlining, which are characteristic of obligate parasites, *P. ramosa* maintains an unusually large and diverse repertoire of about 40 CLPs, possibly resulting from frequent duplication events. The observed transposase activity, while potentially linked to gene diversification, raises intriguing questions about its role in the long-term coevolutionary history with *Daphnia*. Future investigations with a broader genome sampling may help to understand structural variations and genomic adaptations in this host–parasite system, and consequently advancing our understanding of the coevolution between *P. ramosa* and its host and possibly with other coevolving host-parasite systems.

## Supplementary Material

jkag091_Supplementary_Data

## Data Availability

Raw data are deposited at the NCBI SRA database, while the assembled genome as well as the predicted set of protein sequences are available at NCBI GenBank (PRJNA1064691, PRJNA1064693, PRJNA1251300, PRJNA1251303, and PRJNA1251842) and at 10.6084/m9.figshare.25163996. Scripts are available at https://github.com/AThivolle/Thivolle_et_al_2026/. Supplemental data are also available at GSA FigShare: https://doi.org/10.25387/g3.31803640. Supplemental material available at G3 online.
