# Supplementary material for: Genomic insights into antagonistic coevolution: collagen-like protein expansion and genome plasticity in the Daphnia parasite Pasteuria ramosa
Source: G3 (Bethesda). 2026 Apr 11;16(6):jkag091. doi: 10.1093/g3journal/jkag091 (PMC13233619; doi:10.1093/g3journal/jkag091)
Supplement: jkag091_Supplementary_Data [file jkag091_supplementary_data.zip › Supplementary_File_1_G3-2026-406704.docx]

**Supplementary File 1:**

**Comparative genomic between one *P. ramosa* isolate and the rest of the species present on the *Bacillota* tree:**

*P. ramosa* and *P. penetrans* exhibit the lowest BUSCO score in the *Bacillota* phylum (81.2 % and 80.3%, firmicutes_odb10, Figure1B), missing 38 and 39 highly conserved genes of the *Bacillota* phylum, respectively. Thirty-two of these genes are missing from both genomes. Fifteen of these missing genes are involved in translation, ribosomal structure, and biogenesis (Supplementary File Table 1). When comparing the Cluster of Orthologous Groups (COG) genome annotations between *P. ramosa, P. penetrans,* and *Thermoactinomyces spp.,* the *Pasteuria* genomes show distinct reductions, primarily in functions related to amino acid, nucleotide, lipid metabolism, and transport (Supplementary File Table 2). In contrast, translation, replication machinery, and repair functions are unaffected. The core machinery for translation and replication is maintained, but the loss of accessory or regulatory genes (the 15 missing BUSCOs) leads to the low score and reflects a streamlined, but functional, system.

| busco ID | Hypothetical function | COG | *P. ramosa (missing Y/N)* | *P. penetrans (missing Y/N)* |
| --- | --- | --- | --- | --- |
| 101717at1239 | DNA-binding, RecF | L/D/V | Y | Y |
| 160396at1239 | Phosphoribosylformylglycinamidine cyclo-ligase | F/H/O/E | Y | Y |
| 171270at1239 | GTPase Obg | J/D/L | Y | Y |
| 187831at1239 | Aspartate carbamoyltransferase | F/E/K | Y | Y |
| 201740at1239 | Peptide chain release factor 2 | J/O | Y | Y |
| 206314at1239 | S-adenosylmethionine | J | Y | Y |
| 249580at1239 | Rod shape-determining protein MreC | D/N/W | Y | Y |
| 264693at1239 | Ribosomal protein L11 methyltransferase | L/I/Q/K | Y | Y |
| 282217at1239 | Winged helix-like DNA-binding domain superfamily | K/J | Y | Y |
| 282919at1239 | RNA-binding S4 domain | J/D/M | Y | Y |
| 284806at1239 | tRNA (cytidine(34)-2'-O)-methyltransferase | J/L/H | Y | Y |
| 297071at1239 | tRNA(Met) cytidine acetate ligase | H/M/V | Y | Y |
| 300181at1239 | SsrA-binding protein | O | Y | Y |
| 300984at1239 | Transcriptional repressor NrdR | F/K/L/X | Y | Y |
| 317813at1239 | 16S rRNA (Guanine(966)-N(2))-methyltransferase RsmD | L/J/I/H | Y | Y |
| 325820at1239 | DNA recombination-mediator protein A | K/L/D | Y | Y |
| 325844at1239 | Methyltransferase small domain | J/L/H/I | Y | Y |
| 339746at1239 | Ribosome hibernation promoting factor | J/L/K/T | Y | Y |
| 340718at1239 | Heat shock protein Hsp33 | O | Y | Y |
| 344098at1239 | RNA-binding protein | J | Y | Y |
| 354448at1239 | 5-(carboxyamino)imidazole ribonucleotide mutase | F/T/K | Y | Y |
| 369764at1239 | RNA methyltransferase RlmH | J | Y | Y |
| 371313at1239 | Gcp-like domain | J/O/G | Y | Y |
| 37166at1239 | GTP-binding protein | T/J/P | Y | Y |
| 375963at1239 | IreB regulatory phosphoprotein |  | Y | Y |
| 378469at1239 | Dephospho-CoA kinase | H/L/M | Y | Y |
| 382094at1239 | Phosphoribosyltransferase domain | F/T/E | Y | Y |
| 382628at1239 | RNA-binding S4 domain | J/M/D | Y | Y |
| 386385at1239 | Ribosome maturation factor RimP | J | Y | Y |
| 387530at1239 | Segregation and condensation protein A | L/H/C | Y | Y |
| 390248at1239 | Protein of unknown function DUF951 |  | Y | Y |
| 391414at1239 | DNA-binding protein | K/X | Y | Y |
| 405267at1239 | RNA-binding S4 domain | J/M/D | Y | Y |
| 52076at1239 | Mur ligase, C-terminal | M/E/H | Y | Y |
| 66991at1239 | tRNA-guanine(15) transglycosylase-like | J/G | Y | Y |
| 71141at1239 | Dihydroorotase | F/Q/G | Y | Y |
| 7613at1239 | DNA-directed DNA polymerase | L/J/E | Y | Y |
| 9650at1239 | SMCs flexible hinge | L/D | Y | Y |
| 16908at1239 | excinuclease ABC subunit B | L/K/V/O | N | Y |
| 197856at1239 | Release factor glutamine methyltransferase | J/H/I/L | N | Y |
| 205447at1239 | DHHA1 domain | J/L/F/T | N | Y |
| 286413at1239 | Metallo-beta-lactamase | M/J/P | N | Y |
| 29577at1239 | Helicase, C-terminal | L/K/V/J | N | Y |
| 334050at1239 | Endoribonuclease YbeY | J/H/G/I | N | Y |
| 350271at1239 | Mini-ribonuclease 3 | K/J | N | Y |

Supplementary File Table 1: BUSCO genes missing in *P. ramosa* and functions

| COG categories | | *P. ramosa* | | *P. penetrans* | | *Thermoactinomyces sp.* | |
| --- | --- | --- | --- | --- | --- | --- | --- |
|  |  | Number | % | Number | % | Number | % |
| A | **RNA processing and modification** | 0 | 0.0 | 0 | 0.0 | 0 | 0.0 |
| B | **Chromatin Structure and dynamics** | 0 | 0.0 | 0 | 0.0 | 0 | 0.0 |
| C | **Energy production and conversion** | 54 | 6.0 | 57 | 6.2 | 115 | 5.0 |
| D | **Cell cycle control and mitosis** | 22 | 2.4 | 26 | 2.8 | 36 | 1.6 |
| E | **Amino Acid metabolism and transport** | 51 | 5.7 | 58 | 6.3 | 195 | 8.4 |
| F | **Nucleotide metabolism and transport** | 34 | 3.8 | 38 | 4.1 | 87 | 3.8 |
| G | **Carbohydrate metabolism and transport** | 29 | 3.2 | 30 | 3.3 | 92 | 4.0 |
| H | **Coenzyme metabolism** | 51 | 5.7 | 61 | 6.6 | 106 | 4.6 |
| I | **Lipid metabolism** | 34 | 3.8 | 32 | 3.5 | 98 | 4.2 |
| J | **Translation** | 129 | 14.4 | 145 | 15.7 | 171 | 7.4 |
| K | **Transcription** | 45 | 5.0 | 45 | 4.9 | 152 | 6.6 |
| L | **Replication and repair** | 127 | 14.1 | 105 | 11.4 | 171 | 7.4 |
| M | **Cell wall/membrane/envelop biogenesis** | 64 | 7.1 | 60 | 6.5 | 153 | 6.6 |
| N | **Cell motility** | 4 | 0.4 | 5 | 0.5 | 5 | 0.2 |
| O | **Post-translational modification, protein turnover, chaperone functions** | 43 | 4.8 | 44 | 4.8 | 86 | 3.7 |
| P | **Inorganic ion transport and metabolism** | 30 | 3.3 | 29 | 3.1 | 125 | 5.4 |
| Q | **Secondary Structure** | 10 | 1.1 | 6 | 0.7 | 87 | 3.8 |
| R | **General Functional Prediction only** | 0 | 0.0 | 0 | 0.0 | 0 | 0.0 |
| S | **Function Unknown** | 147 | 16.4 | 160 | 17.4 | 491 | 21.2 |
| T | **Signal Transduction** | 16 | 1.8 | 17 | 1.8 | 91 | 3.9 |
| U | **Intracellular trafficking and secretion** | 19 | 2.1 | 17 | 1.8 | 33 | 1.4 |
| Y | **Nuclear structure** | 0 | 0.0 | 0 | 0.0 | 0 | 0.0 |
| Z | **Cytoskeleton** | 1 | 0.1 | 1 | 0.1 | 1 | 0.0 |
| - | **not annotated** | 19 | 2.1 | 20 | 2.2 | 117 | 5.1 |
| V | **Defense mechanisms** | 7 | 0.8 | 6 | 0.7 | 60 | 2.6 |
| W | **Extracellular structures** | 0 | 0.0 | 0 | 0.0 | 1 | 0.0 |
| Number of genes annotated | | 898 | | 921 | | 2316 | |

Supplementary File Table 2: COGs functional annotation of *P. ramosa*, *P. penetrans* and *Thermoactinomyces sp..*

The number indicate the number in each category.

Percentage is the ratio between the annotation per category by the total number of proteins annotated.

**Comparative genomic between the five *P. ramosa* isolates:**

Most of the genes are shared by the five isolates. But, among the 20 genes exclusive to the *Daphnia Daphnia* infective parasites, three encode ABC (ATP-Binding Cassette) permease transporters. ABC transporters are widely distributed in the bacteria world, one of the most ubiquitous proteins (Choi and Ford 2021; Akhtar and Turner 2022). ABC transporters are known for their versatility, playing significant roles in both nutrient acquisition and detoxification processes. They can operate bi-directionally, facilitating the uptake of essential nutrients, amino acids, and other organic compounds. Concurrently, these transporters can act as efflux pumps, expelling toxic substances and xenobiotics from the cell, thereby contributing to the organism's ability to survive in various environments (Akhtar and Turner 2022). These specific transporters in the *Daphnia Daphnia* infectors are part of the ABC-4 Integral Membrane family. This protein seems relatively conserved in the *Bacillota* tree and might have been conserved in the *Daphnia Daphnia* infectors while the *Pasteuria* infecting *D. magna* lost it.

**References**

Akhtar AA, Turner DPJ. 2022. The role of bacterial ATP-binding cassette (ABC) transporters in pathogenesis and virulence: Therapeutic and vaccine potential. Microb Pathog. 171:105734. https://doi.org/10.1016/j.micpath.2022.105734

Choi CC, Ford RC. 2021. ATP binding cassette importers in eukaryotic organisms. Biol Rev. 96(4):1318–1330. https://doi.org/10.1111/brv.12702
