# Supplementary material for: Genomic insights into antagonistic coevolution: collagen-like protein expansion and genome plasticity in the Daphnia parasite Pasteuria ramosa
Source: G3 (Bethesda). 2026 Apr 11;16(6):jkag091. doi: 10.1093/g3journal/jkag091 (PMC13233619; doi:10.1093/g3journal/jkag091)
Supplement: jkag091_Supplementary_Data [file jkag091_supplementary_data.zip › Supplementary_File_2_G3-2026-406704.docx]

**Supplementary File 2**

Methods:

All the entries from protein NCBI database containing “collagen-like” in the taxon Thermoactinomycetaceae have been downloaded (accessed 03.03.2026). They have been compared to the number of genome present in genome NCBI database for the same taxon (accessed the same day).

Results:

|  | Genomes in NCBI | Genomes containing CLPs | Total numbers of CLPs |
| --- | --- | --- | --- |
| **Thermoactinomycetaceae** | 113 | 11 | 187 |
| environmental samples | 4 | 0 | 0 |
| unclassified Thermoactinomycetaceae | 4 | 1 | 1 |
| Baia | 1 | 1 | 34 |
| Croceifilum | 1 | 1 | 10 |
| Desmospora | 3 | 0 | 0 |
| Geothermomicrobium | 0 | 0 | 0 |
| Hazenella | 1 | 0 | 0 |
| Kroppenstedtia | 10 | 1 | 1 |
| Laceyella | 11 | 1 | 3 |
| Lihuaxuella | 2 | 0 | 0 |
| Marininema | 2 | 0 | 0 |
| Marinithermofilum | 1 | 0 | 0 |
| Mechercharimyces | 1 | 0 | 0 |
| Melghirimyces | 5 | 0 | 0 |
| Novibacillus | 4 | 0 | 0 |
| Paenactinomyces | 3 | 0 | 0 |
| Paludifilum | 1 | 0 | 0 |
| Planifilum | 9 | 0 | 0 |
| Polycladomyces | 3 | 0 | 0 |
| Polycladospora | 2 | 1 | 1 |
| Risungbinella | 2 | 0 | 0 |
| Salinithrix | 1 | 0 | 0 |
| Seinonella | 1 | 0 | 0 |
| Shimazuella | 3 | 3 | 9 |
| Staphylospora | 1 | 0 | 0 |
| Thermoactinomyces | 35 | 2 | 128 |
| Thermoflavimicrobium | 2 | 0 | 0 |
|  |  |  |  |

**Supplementary File 2 Table1**

A limited number of genomes in the taxon *Thermoactinomycetacea* are presenting collagen-like proteins, only 11 out of 113 (Sup File S2 Table1). Most of these species are presenting few CLPs genes per genomes, but 4 species are presenting more than 10 CLPs per genome (File S2 Table 2). They *are Baia soyae, Croceifilum oryzae, Thermoactinomyces sp. DSM 45891* and *Thermoactinomyces sp. DSM 45892.*

| Genus | Species | Number of CLPs |
| --- | --- | --- |
| Baia | Baia soyae | 34 |
| Croceifilum | Croceifilum oryzae | 10 |
| Kroppenstedtia | Kroppenstedtia sanguinis | 1 |
| Laceyella | Laceyella putida | 3 |
| Polycladospora | Polycladospora coralii | 1 |
| Shimazuella | Shimazuella alba | 2 |
| Shimazuella | Shimazuella kribbensis | 2 |
| Shimazuella | Shimazuella soli | 5 |
| Unclassified Thermoactinomycetaceae | Thermoactinomyces | 1 |
| Thermoactinomyces | Thermoactinomyces sp. DSM 45891 | 55 |
| Thermoactinomyces | Thermoactinomyces sp. DSM 45892 | 73 |

**Supplementary File 2 Table2**
